# Protective immunity induced by a novel P1 adhesin C-terminal anchored mRNA vaccine against *Mycoplasma pneumoniae* infection in BALB/c mice

**DOI:** 10.1128/spectrum.02140-24

**Published:** 2025-01-20

**Authors:** Qilin Zeng, Peiyuan Sun, Weiwei Li, Yuanyuan Tang, Yuxuan Hu, Jun Zhou, Yanxia Zhou, Liesong Chen, Wu Yimou

**Affiliations:** 1 Hunan Province Cooperative Innovation Center for Molecular Target New Drug Study, Hengyang Medical College, Institute of Pathogenic Biology, University of South China, Hengyang, China; 2 Department of Clinical Laboratory, The Second People’s Hospital of Foshan, Foshan, China; 3 Department of Critical Care Medicine, Xiangya Hospital, Central South University 159374 , Changsha, China; University of North Dakota, Grand Forks, North Dakota, USA

**Keywords:** *Mycoplasma pneumoniae*, P1 adhesin, dominant antigenic epitope, mRNA vaccine, lipid nanoparticle, protective immunity

## Abstract

*Mycoplasma pneumoniae* (Mp), a unique pathogen devoid of a cell wall, is naturally impervious to penicillin antibiotics. This bacterium is the causative agent of *M. pneumoniae* pneumonia, an acute pulmonary affliction marked by interstitial lung damage. Non-macrolide medications may have potential adverse effects on the developmental trajectory of children, thereby establishing macrolides as the preferred treatment for *M. pneumoniae* in pediatric patients. However, the emergence of macrolide-resistant and multidrug-resistant strains of *M. pneumoniae* presents significant challenges to clinical management and public health. Vaccines, particularly those based on mRNA technology, are regarded as a promising avenue for preventing and controlling *M. pneumoniae* infections due to their inherent safety, immunogenicity, and adaptability. Our research delves into the P1 adhesin of *M. pneumoniae*, a protein that binds to host cell receptors with its immunodominant epitopes located at the carboxyl terminus, known to provoke robust immune responses and pulmonary inflammation. We have developed an mRNA vaccine harnessing this dominant antigenic epitope and assessed its protective immunity in BALB/c mice against *M. pneumoniae* infection. The vaccine elicited potent humoral and cellular immune responses, effectively diminishing inflammation. It notably decreased IL-6 levels in the lungs of infected mice and concurrently elevated IL-4, IL-10, and IFN-γ levels post-immunization. The vaccine also reduced pathological changes in the lungs and the *M. pneumoniae* DNA copy numbers in the infected animals. Collectively, these findings underscore the mRNA vaccine’s remarkable immunogenicity and protective potential against *M. pneumoniae* infections, offering valuable insights for the development of mRNA vaccines targeting mycoplasma infections.

**IMPORTANCE:**

*M. pneumoniae*, a bacteria without a cell wall, is known for causing pneumonia and is resistant to penicillin. The increasing prevalence of macrolide-resistant strains has complicated treatment options, emphasizing the need for new strategies. Our research explores an mRNA vaccine candidate that targets the P1 adhesin of *M. pneumoniae*, a protein critical for the bacteria’s interaction with host cells. In a mouse model, this vaccine has shown potential by inducing immune responses and suggesting a possible reduction in inflammation, as indicated by changes in cytokine levels and lung pathology. While further research is required, the vaccine’s preliminary results hint at a potential new direction in managing mycoplasma infections, offering a promising avenue for future therapeutic development. This study contributes to the ongoing search for effective preventive measures against *M. pneumoniae*.

## INTRODUCTION

*Mycoplasmas* are the smallest prokaryotic microorganisms that lack a cell wall, exhibit high polymorphism, and can grow in lifeless culture media. Adhesion is crucial for *Mycoplasma pneumoniae* colonization and infection, with most pathogenic *M. pneumoniae* species losing their virulence when their adhesive capacity is diminished (1). *M. pneumoniae* adhesion organelles, including P1 adhesin and associated lipoproteins, assist in biofilm formation (2–5), allowing the pathogen to colonize and invade host cell surfaces, leading to immune evasion. P1 adhesin, a trypsin-sensitive protein located at the terminal structure of *M. pneumoniae*, directly binds to specific receptors on host cell membranes, playing a critical role in adhesion and gliding motility. Studies have shown that antibodies against P1 can specifically block the binding of *M. pneumoniae* to animal epithelial cells and slow the gliding movement of *M. pneumoniae* (6). Research indicates that P1 adhesin exhibits high immunoreactivity; monoclonal anti-P1 antibodies can inhibit adhesion, and the absence of P1 adhesin prevents *M. pneumoniae* attachment (7). The interaction between *M. pneumoniae* adhesion organelles and the host respiratory epithelium induces cytoskeletal rearrangements in host cells, facilitating intracellular delivery of the pathogen (1, 8–10).

Several mycoplasma species can colonize the human body without causing disease. However, only *M. pneumoniae* is known to cause respiratory diseases. *M. pneumoniae* is a leading pathogen responsible for community-acquired pneumonia (CAP) in both children and adults (11). The severity of *M. pneumoniae*-induced pneumonia is typically mild; however, in severe cases, it can pose a life-threatening risk. In addition to encephalitis, this infection has the potential to cause neurological complications such as Guillain-Barré syndrome—an immune-mediated demyelinating polyneuropathy (12, 13)—as well as other disorders including acute disseminated encephalomyelitis, transverse myelitis, and opsoclonus-myoclonus syndrome. These manifestations underscore the significant health hazards associated with *M. pneumoniae* infections (14–16). In China, the incidence of *M. pneumoniae* pneumonia (MPP) is relatively high, accounting for 19.2% of adult CAP cases (17). Among children and adolescents, the incidence of CAP ranges from 10% to 40% (18). The primary clinical treatment for MPP is macrolide antibiotics. However, the global prevalence of macrolide-resistant or multidrug-resistant *M. pneumoniae* strains is rising annually, presenting significant challenges to clinical treatment (19–23).

Research has shown that monoclonal antibodies targeting amino acids 1,107–1,518 of the P1 adhesin can react with HEp-2 cells, effectively inhibiting *M. pneumoniae* adhesion to host cells and significantly reducing the number of *M. pneumoniae* microcolonies on these cells, thereby mitigating cellular damage to a certain extent (24). Zhu et al. constructed a recombinant P1C fragment (P1C) and discovered that this fragment could adhere to HeLa cells. In addition, anti-P1 antibodies were able to effectively block *M. pneumoniae* adhesion to HeLa cells, demonstrating the excellent immunogenicity and immune reactivity of P1C. In BALB/c mice, intramuscular or intranasal administration of a DNA vaccine encoding the C-terminal region (P1C) of *M. pneumoniae* P1 adhesin (amino acids 1,125–1,395) resulted in significantly elevated levels of IgG (including the IgG1, IgG2a, and IgG2b subclasses) and cytokines (IL-4, IL10, and IFN-γ), indicating protective effects against *M. pneumoniae* infection. Furthermore, studies have shown that the recombinant purified P1 adhesin (amino acids 1,160–1,521) reacts well with individual and combined positive sera and anti-histidine antibodies (25). These findings collectively suggest that the C-terminal region of P1 adhesin contains adhesion epitopes and possesses strong immunogenicity and immune reactivity (26). Despite the promising research on P1 adhesin and its potential for vaccine development, no highly effective vaccine for preventing *M. pneumoniae* infection in humans is currently available.

Common types of vaccines on the market include DNA vaccines, subunit vaccines, live vaccines, and mRNA vaccines. Compared to traditional vaccines, mRNA vaccines offer several advantages in terms of safety, immunogenicity, and flexibility (27, 28). In addition, they demonstrate distinct cost characteristics in both research and production. Traditional vaccines typically necessitate intricate biotechnological processes such as cell culture, virus inactivation, or attenuation which can result in elevated production costs and longer production cycles (29). By contrast, the production process for mRNA vaccines is comparatively straightforward and enables rapid generation of substantial vaccine quantities through *in vitro* transcription (IVT), thereby reducing unit costs and production time. mRNA vaccines are synthesized via IVT using DNA templates containing antigen-coding sequences. The resulting mRNA molecules enter the host cells’ cytoplasm through endocytosis. Subsequently, a portion of the mRNA binds to ribosomes within host cells and undergoes successful translation into antigenic proteins. These proteins can be degraded by proteasomes into antigen peptides, which are then presented to cytotoxic T lymphocytes (CTLs) via the major histocompatibility complex (MHC) I pathway. Alternatively, they can be released from host cells and taken up by dendritic cells (DCs). The antigens are subsequently degraded and presented to helper T cells and B cells through the MHC II pathway. In addition, B cells can recognize the released antigenic proteins (28). Furthermore, mRNA vaccines have intrinsic adjuvant effects. The IVT products of mRNA can be recognized by various pattern recognition receptors (PRRs) (30, 31). Although mRNA vaccine research began as early as the early 1990s, their initial use was limited due to the instability of mRNA, which is susceptible to degradation by ribonucleases. Over the past few decades, significant advancements have been made, and mRNA vaccines are now widely used to prevent infectious diseases and treat cancers. These vaccines have demonstrated remarkable efficacy, as evidenced by their rapid development and deployment during the COVID-19 pandemic. Furthermore, mRNA vaccines are currently being investigated for a variety of infectious diseases, including Zika, HIV, and influenza. In the field of oncology, mRNA vaccines have exhibited promising results in encoding tumor-associated antigens and eliciting immune responses against cancers such as pancreatic cancer (32–35). They have shown substantial progress in inducing robust and lasting immune responses against viral and other pathogenic infections in both animals and humans. Studies have demonstrated that mRNA vaccines can induce safe and durable immune responses in various infection models, highlighting their potential. Given these advancements, developing a mRNA vaccine for *M. pneumoniae* to control *M. pneumoniae* infections is highly feasible. The flexibility and efficacy of mRNA vaccines make them an excellent candidate for addressing the challenges posed by *M. pneumoniae*, particularly in light of the rising prevalence of drug-resistant strains (36, 37).

Building on previous research and leveraging bioinformatics analyses, we selected the dominant antigenic epitope of the P1 adhesin C-terminus (amino acids 1079–1518) for designing a mRNA vaccine against *M. pneumoniae*. These analyses included predictions of transmembrane domains, secondary and tertiary structural features, functional domain analysis, and immunogenicity assessments. In this study, we constructed a cloning vector, pGEM-3zf(+)/RP1C_1079–1518_, and investigated the immunogenicity and immune protection conferred by intramuscular injection of this vector against intranasal *M. pneumoniae* infection in BALB/c mice. The results of this research will provide experimental evidence for the development of a mRNA vaccine against *M. pneumoniae* infection.

## MATERIALS AND METHODS

### Cells, plasmid vectors, bacterial strains, and mice

A549 human non-small-cell lung cancer cells were cultured in Dulbecco’s modified Eagle medium (DMEM) medium (Thermo Fisher Scientific, USA) supplemented with 10% fetal bovine serum (FBS) at 37℃ with 5% CO_2_. The plasmid vectors used in this study were pGEM-3zf(+)/RP1C_1079–1518_ for cloning and pET-28a(+)/RP1C_1079–1518_ for expression. The genomic DNA of the *M. pneumoniae* M129 standard strain (ATCC 29342) was used as a template to design primers. The selected gene sequences were amplified and identified through PCR, restriction enzyme digestion, and sequencing. The construction of the eukaryotic cloning vector and prokaryotic expression vector was outsourced to Sangon Biotech (Shanghai, China), where synthesis and culture in the LB medium were conducted. The *M. pneumoniae* M129 strain (ATCC 29342) used in this study is maintained at the Institute of Pathogenic Biology, University of South China. It was cultured in the pleuropneumonia-like organism (PPLO) medium (BD Biosciences) containing 20% FBS, 0.25% glucose, 0.25% yeast extract (Oxoid), 1,000 U/mL penicillin G, and 0.005% phenol red. The pH was adjusted to 7.9, and the culture was incubated at 37℃ for approximately 5–7 days until the medium color changed from red to orange. Specific pathogen-free, 6-week-old female BALB/c mice were purchased from Hunan SJA Laboratory Animal Co., Ltd. All animal research procedures were conducted in Animal Biosafety Level 3 (ABSL-3) facilities.

### Bioinformatics analysis, sequence selection, and vaccine design of *M.pneumoniae* P1 adhesin

The gene sequence of the P1 adhesin from the *M. pneumoniae* M129 reference strain was obtained from NCBI (http://www.ncbi.nlm.nih.gov/). The C-terminal domain of the P1 adhesin was identified using Uniprot (https://www.uniprot.org/). SMART (http://smart.embl-heidelberg.de/) was used to predict the functional domains within the C-terminal sequence of the P1 adhesin. Antigenicity predictions were performed using ANTIGENpro (http://scratch.proteomics.ics.uci.edu/) and VaxiJen v2.0. Allergenicity was assessed using AllergenFP v1.0 and AllerTOP v2.0. For the vaccine design, the 5′UTR sequences of human α-globin A1 (HBA1) and human hemoglobin A2 (HBA2) were selected and included the Kozak consensus sequence for translation initiation in eukaryotic cells, followed by a secretion signal peptide. The C-terminal adhesion epitope of P1 adhesin, spanning amino acids 1079–1518, was selected as the mRNA-coding region, with the stop codon TGATGA. The 3′UTR consisted of sequences from human AES/TLE5 and mitochondrial 12sRNA (mtRNR1), with mutations at amino acid positions 1098 and 1159 from TGA to TGG. The secondary structure of the protein was predicted using SOPMA. Various physicochemical parameters of the vaccine, such as aliphatic index, extinction coefficient, instability index, theoretical pI, and molecular weight, were analyzed using ProtParam (https://web.expasy.org/protparam). The tertiary structure of the protein was modeled using SWISS-Model (http://swissmodel.expasy.org/). The optimal and minimal free energy required for the mRNA secondary structure was predicted using RNAfold WebServer (http://rna.tbi.univie.ac.at/cgi-bin/RNAWebSuite/RNAfold.cgi).

### Expression and linearization of P1C_1079–1518_

The recombinant pET-28a(+)/RP1C1079–1518 expression vector was transformed into Escherichia coli BL21(DE3) and cultured. When the OD_600_ reached 0.8, 1 mM IPTG was added and expression was induced at 16°C, 180 rpm for 6 h. The protein was expressed and purified using ultrasonication, SDS-polyacrylamide gel electrophoresis (PAGE) analysis, Ni-NTA affinity chromatography, and detox-gel chromatography to remove endotoxins. The protein concentration was determined using a BCA assay kit. The pGEM-3zf(+)/RP1C1079–1518 cloning vector was transformed into *Escherichia coli* JM109 and cultured. When the OD_600_ reached 0.8, bacterial cultures were collected, and DNA was extracted using a bacterial DNA extraction kit (Qiagen, Hilden, Germany). The circular plasmid DNA was linearized using the restriction enzyme BamHI, and the linearized DNA was purified using the phenol-chloroform method.

### Preparation and encapsulation of LNP and mRNA

Lipid nanoparticles (LNPs) were prepared with a molar ratio of DOTAP:DOPE:DSPE-PEG2000 (mol/mol) =50:50:1, where all lipids were purchased from MedChemExpress (Monmouth Junction, NJ, USA). LNP/mRNA complexes were prepared at a specified N:P molar ratio (N representing the total molar amount of nitrogen in DOTAP, and P representing the total molar amount of phosphorus in mRNA). Assuming an average molar mass of 330 Da per nucleotide in RNA, the molar ratio between DOTAP and RNA was calculated. mRNA synthesis followed the protocol provided by the HyperScribe All in One mRNA Synthesis Kit Plus 1 (APExBIO Technology LLC, Houston, Texas, USA), with T7 RNA polymerase based on the linearized plasmid pGEM-3zf(+)/RP1_1079–1518_. The synthesized mRNA was purified using the phenol-chloroform extraction method. The resultant mRNA included an optimal anti-reverse 3′-O-Me-m7G(5′)ppp(5′)G cap analog, enhancing its stability. The addition of 5mCTP and ψUTP modifications significantly reduced the immunogenicity of the *in vitro* transcribed mRNA and improved its stability and translation efficiency. The purified mRNA was stored at −80°C for further use. mRNA was encapsulated in LNPs through a self-assembly process. The pre-prepared aqueous mRNA solution and lipid nanoparticle solution dissolved in ethanol were transferred to a microfluidic device, where the aqueous phase and organic phase were merged at a 3:1 ratio. The mixture was thoroughly blended using a magnetic stirrer, encapsulating the mRNA within the LNPs. The final LNP-mRNA product was obtained by centrifugation using a 10 kDa ultrafiltration tube (Millipore, Billerica, MA, USA) to concentrate the sample. The size and zeta potential of the LNP and LNP-mRNA particles were measured using a Malvern Zetasizer Nano ZS90 nanoparticle size analyzer (Malvern Panalytical, Westborough, MA, USA). Finally, the morphology of the particles was observed using transmission electron microscopy (TEM).

### mRNA transfection

A549 cells were seeded at a density of 1 × 10^6^ cells per well in a sterile 6-well cell culture plate with DMEM medium supplemented with 10% FBS. The cells were cultured at 37°C with 5% CO_2_ until they reached 80% confluence. The medium was discarded, and the cells were washed twice with 2 mL phosphate-buffered saline (PBS) per wash. After discarding the PBS, 500 µL of Opti-MEM medium was added to each well. The prepared LNP-mRNA (mRNA = 2.5 µg per well) was transfected into the cells using Lipofectamine 3000 transfection reagent (Thermo Fisher Scientific, USA). The plate was incubated at 37°C with 5% CO_2_. 6 hours post-transfection, 500 µL of DMEM medium containing 10% FBS was added to each well to supplement the medium. 48 hours after transfection, the cells were collected to extract proteins.

### Cytotoxicity assay

The cytotoxicity of LNPs prepared with different N/P ratios was assessed using the Cell Counting Kit-8 (CCK-8) following the manufacturer’s instructions. This assay was conducted to determine the safe and effective dosage range of LNPs, avoiding excessive toxicity that could lead to cell damage. A549 cells were seeded at a density of 1 × 10^6^ cells per well in a 96-well plate and then treated with the prepared LNPs. After 24 hours of incubation, 10 µL of CCK-8 solution was added to each well. The plate was incubated at 37°C for 4 hours, and the absorbance was measured at 450 nm to determine cell viability.

### Western blot

48 hours after transfection, cells transfected with LNP-mRNA were collected for Western blot analysis to detect the expression of RP1C_1079–1518_. The cells were lysed in RIPA buffer containing PMSF protease inhibitor at 4°C. The lysate was then subjected to sonication at 20% power with a cycle of 3 seconds on and 5 seconds off, repeated three times on ice. The samples were centrifuged at 14,000 rcf for 10 minutes at 4℃, and the supernatant was collected. One-fourth volume of 5 × SDS-PAGE loading buffer was added to the supernatant, mixed thoroughly, and the proteins were denatured by incubating in a 100°C water bath for 10 minutes. The samples were then subjected to SDS-PAGE and transferred onto a polyvinylidene difluoride (PVDF) membrane. The membrane was blocked in 5% non-fat milk for 2 hours. After blocking, the membrane was probed with primary antibodies against *M. pneumoniae* and β-actin (Abcam, UK). Following primary antibody incubation, the membrane was incubated with HRP-conjugated secondary antibodies. Protein bands were visualized using chemiluminescence detection methods.

### Mice immunization, vaccine biosafety, and protective efficacy

Six-week-old female BALB/c mice were randomly divided into four groups (10 mice per group): two immunization groups (LNP-mRNA and RP1C1079–1518) and two negative control groups (LNP-mRNA UTRs and PBS). The mice were weighed and then intramuscularly injected on days 0, 14, 28, and 42. Mice in the immunization groups were injected with 15 µg of LNP-mRNA or 15 µg of RP1C1079–1518. The negative control groups received either LNP-mRNA UTRs without an open reading frame or PBS. Before each immunization on days 14, 28, and 42, blood was collected from the tail vein using a blade. The blood was allowed to clot for 30 minutes at 37°C and then centrifuged at 6,000 rpm for 10 minutes at 4°C. The serum was collected and stored at −20°C for subsequent enzyme-linked immunosorbent assay (ELISA) to detect the level of ags-specific IgG and evaluate the humoral immune response induced by the vaccine. Two weeks after the fourth immunization (day 56), five mice from each group were randomly selected for retro-orbital blood collection and spleen extraction. Clinical laboratory tests were conducted, including liver function, kidney function, blood glucose, blood lipids, cardiac enzymes, and pancreatic biomarkers. Flow cytometry was used to evaluate T-lymphocyte responses and assess vaccine safety. The remaining five mice in each group were intranasally challenged, which was performed under anesthesia. Specifically, mice were anesthetized using isoflurane applied via a cotton swab within a sealed box. Once the mice were observed to be adequately anesthetized, 50 µL of 1 × 10^5^ CFUs of *M. pneumoniae* was administered via the intranasal route without delay on days 56 and 57. Fourteen days post-infection, the mice were euthanized, and lung tissues were aseptically isolated. The lung tissues were subjected to hematoxylin and eosin (H&E) staining, cytokine expression analysis, and DNA extraction for qPCR to determine *M. pneumoniae* DNA copy numbers. These analyses were used to evaluate the vaccine-induced protective immune response against infection.

### Spleen lymphocyte proliferation assay

Mice from the negative control group were euthanized, and their spleens were harvested. Subsequently, a splenocyte suspension was prepared from the excised spleens. The density of the spleen cell suspensions was calculated using a hemocytometer. The spleen cells were then diluted to an appropriate concentration with RPMI-1640 medium. Samples from each diluted spleen cell suspension were seeded at a density of approximately 2 × 10^6^ cells per well in a 96-well cell culture plate. Each sample was plated in quadruplicate to establish the following experimental groups: the control stimulation group, which included wells with PBS and LNP-mRNA_UTRS_, and the vaccine stimulation group, which included wells with RP1C_1079–1518_ and LNP-mRNA. For the vaccine stimulation group, 10 µg of RP1C_1079–1518_ protein and LNP-mRNA were added separately to each well to stimulate the spleen cells. In the control stimulation group, 10 µg of LNP-mRNA_UTRS_ was added to each well to stimulate the spleen cells, and an equivalent volume of PBS was added to the wells designated for the PBS control. The plates were thoroughly mixed and incubated in a 37°C incubator with 5% CO_2_. After 48 hours of incubation, 10 µL of CCK-8 reagent was added to each well, and the plates were mixed thoroughly again. The plates were then incubated in the dark at 37°C with 5% CO_2_ for 1–4 hours, monitoring the color change of the solution. The incubation was stopped before the color became too dark to avoid affecting subsequent absorbance measurements. The absorbance at 450 nm was measured using a microplate reader. The stimulation index (SI) was calculated according to the Cell Counting Kit-8 instructions using the formula: SI = (OD value of stimulated group − OD value of blank group)/(OD value of unstimulated group − OD value of blank group).

### Enzyme-linked immunosorbent assay

To detect anti-mycoplasma antibodies in mouse serum, the plates were coated with the endotoxin-free purified P1C1079–1518 antigen for the detection of specific antibodies and was diluted to 10 µg/mL with 1 × coating buffer. Each well of a 96-well ELISA plate was coated with 100 µL of this solution. After washing three times with PBST (PBS containing 0.05% Tween 20), the wells were blocked with 200 µL of 1 × ELISpot blocking buffer per well and incubated at 37°C for 2 hours. Serially diluted serum samples (100 µL per well) were added to the plate and incubated at 37°C for 2 hours. The wells were washed three times, and 100 µL of HRP-conjugated goat anti-mouse IgG (1:10,000, Abcam, USA) was added to each well. The plate was incubated at 37°C for 2 hours. After three washes, 100 µL of TMB substrate was added to each well, and the plate was kept in the dark at 37°C for 15 minutes. The reaction was stopped by adding 100 µL of stop solution to each well. The absorbance was measured at 450 nm using a microplate reader (Tecan, Switzerland). The absorbance value of the PBS negative control group was used as the standard. A sample was considered to have a positive antibody titer if the absorbance value of the experimental group was greater than 2.1 times that of the negative control group. The highest serum dilution that yielded a positive response was recorded as the specific IgG antibody titer. In addition, cytokine levels in the serum, such as IL-4, IL-6, IL-10, and IFN-γ, were measured using the respective ELISA kits (Thermo Fisher Scientific, Waltham, MA, USA).

### Flow cytometry

On day 56 after the first immunization, spleens were harvested from the mice. Spleens were ground and filtered through a 70 µm cell strainer (BD Biosciences, San Jose, CA, USA) using RPMI-1640 medium. The cell suspension was centrifuged at 1,000 rcf for 5 minutes at 4°C. The supernatant was discarded, and the red blood cells in the spleen cell pellet were lysed using red blood cell lysis buffer (Beyotime, Shanghai, China). The lysis was stopped by adding twice the volume of PBS, mixed well, and centrifuged again at 1,000 rcf for 5 minutes at 4°C. The cells were washed once with PBS and resuspended in 300 µL of RPMI-1640 medium containing 10% FBS. For flow cytometry staining, 0.2 µL each of FITC anti-mouse CD3ε antibody, PerCP anti-mouse CD4 antibody, and APC anti-mouse CD8a antibody (BioLegend, San Diego, CA, USA) were added to flow cytometry tubes. PBS was added to each tube to bring the volume to 50 µL. Then, 5 µL of the spleen cell suspension was added to each tube. The tubes were mixed thoroughly and incubated for 30 minutes at 4°C in the dark. The reaction was terminated by adding 200 µL of PBS, and the samples were analyzed using a flow cytometer (BD Biosciences, San Jose, CA, USA).

### Detecting *M. pneumoniae* load and DNA copy numbers

Lung tissue homogenate was prepared by adding pre-cooled saline and prepared in an ice bath at 4℃. The tissue homogenate was then diluted with saline and coated with PPLO plates The coated plates were incubated in an incubator at 37°C for 7 days for colony counting.

DNA was extracted from homogenized mouse lung tissues using the TIANamp Genomic DNA Kit (TIANGEN Biotech, Beijing, China). The extracted DNA was used as a template for quantitative PCR (Q-PCR) amplification of the *M. pneumoniae* p1 adhesin 16S rRNA sequence and mouse β-actin sequence. The gene-specific primers and probe for amplifying the *M. pneumoniae* p1 adhesin 16S rRNA sequence were as follows:

– Forward primer: 5′- CAATGCCATCAACCCGCGCTTAACC-3′
– Reverse primer: 5′- CGTGGTTTGTTGACTGCCACTGCCG-3′

The primers for amplifying the mouse β-actin sequence were as follows:

– Forward primer: 5′- CCTTCCTTCTTGGGTATGGA-3′
– Reverse primer: 5′- ACGGATGTCAACGTCACACT-3′

Q-PCR was performed on a PCR instrument (Roche, Basel, Switzerland) to detect and quantify gene expression levels.

### Histological examination of lung tissue

After euthanizing the mice, the lung tissues were fixed in 4% paraformaldehyde, embedded in paraffin, sectioned continuously, and stained with hematoxylin and eosin (H&E) for histological analysis. Lung pathology was primarily characterized by alveolar wall thickening, inflammatory cell infiltration, and hemorrhage. The extent of inflammation in the trachea, bronchioles, perivascular areas, and lung interstitium was scored based on the method described by Peebles RS et al. (38) with a scoring system of 1–3 points as follows:

a. Peri-tracheal and peri-bronchiolar inflammation: The severity of inflammation was indicated by the thickness of the inflammatory infiltrate around the trachea and bronchioles. Several sections were counted per slide to calculate the average thickness.

– 0 points: No infiltration
– 1 point: Thickness of less than 2 cell layers
– 2 points: Thickness of 3–5 cell layers
– 3 points: Thickness of more than 5 cell layers

b. Perivascular inflammation: The severity was evaluated similarly to peri-tracheal and peri-bronchiolar inflammation.

– 0 points: No infiltration
– 1 point: Thickness of less than 4 cell layers
– 2 points: Thickness of 5–7 cell layers
– 3 points: Thickness of more than 7 cell layers

c. Interstitial inflammation: The inflammation in the alveolar septa was scored as follows:

– 0 points: No infiltration
– 1 point: Presence of inflammatory infiltration without thickening
– 2 points: Marked inflammatory infiltration with mild thickening
– 3 points: Marked inflammatory infiltration with significant thickening

Identification criteria for trachea and blood vessels:

– Trachea: Regular columnar cells with blue nuclei
– Blood vessels: Particularly arteries with a red muscle layer, blue nuclei for both inflammatory and other cells, with inflammatory cells being located outside the structure of the blood vessels, trachea, or interstitium.

Scoring method: Each mouse’s lung tissue was observed under five different fields of view. For each field, three scores were added together to obtain a single field score. The average of these scores was calculated to determine the pathological score for each mouse.

### Animal ethics statement

Six-week-old specific pathogen-free female BALB/c mice were obtained from Hunan SJA Laboratory Animal Co., Ltd. (Changsha, China) and housed at the Animal Experiment Research Center of Nanhua University. All experimental protocols involving animals were approved by the Ethics Committee of the Animal Experiment Research Center of Nanhua University. The handling of mice complied with the National Guidelines for the Care and Use of Laboratory Animals. All animal studies were conducted with approval from the Animal Welfare Committee of Nanhua University (USC) and were performed in accordance with the university’s regulations, with efforts made to minimize animal suffering. This research was approved by the Ethics Committee of Nanhua University and adhered to the principles of the Declaration of Helsinki. Approval number: USC202306PB02.

### Statistical analysis

Statistical analyses were performed using Excel and GraphPad Prism 8.0.2 software. For data with a normal distribution, the Student’s t-test was used to analyze the significance of differences between groups or analyzed for statistical significance using one-way ANOVA for multiple comparisons. For multiple group comparisons, two-way ANOVA was used. All data are presented as mean ± 95% CI. Statistical significance tested by one-way ANOVA test (*P < 0.05; **P < 0.01; ***P < 0.001; ****P < 0.0001). Data that were not analyzed to be normally distributed were analyzed using nonparametric statistical analysis, that is, the Kruskal-Wallis H test.

## RESULTS

### Bioinformatics analysis of P1C_1079–1518_

**Fig 1:**
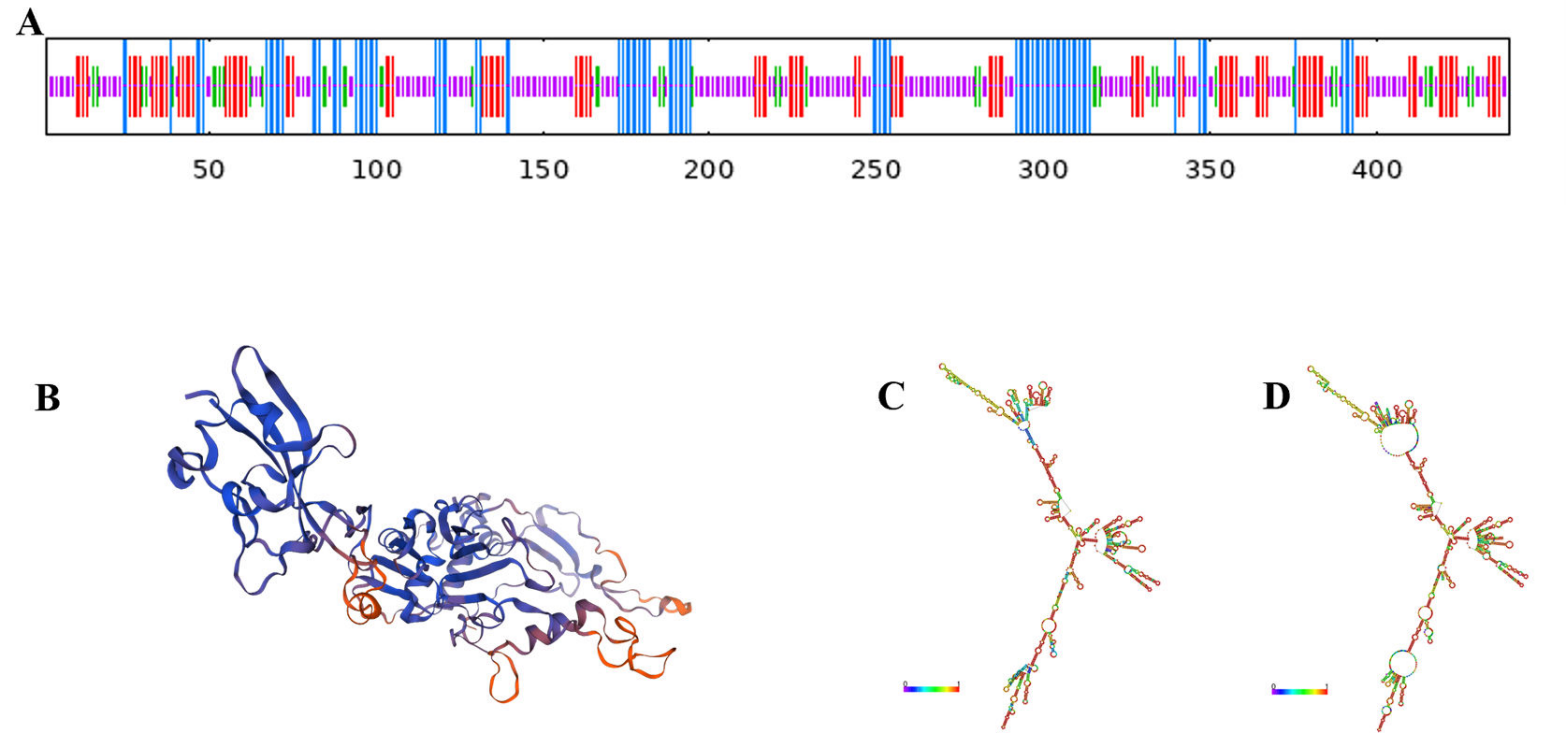
Bioinformatics Analysis of *M. pneumoniae* P1C1079–1518. (A) Prediction of the secondary structure features of P1C1079–1518. (B) Prediction of the tertiary structure of P1C1079–1518. (C) Minimum free energy (MFE) secondary structure of mRNA encoding P1C1079–1518. (D) Centroid secondary structure of mRNA encoding P1C1079–1518.

Using SMART software, we analyzed the C-terminal region of *M. pneumoniae* P1 adhesin and identified numerous functional domains with significant roles. We selected the P1C_1079–1518_ sequence as the dominant antigenic epitope based on high antigenicity, non-allergenicity, a high G + C ratio, and a high ratio of α-helices and random coils in its secondary structure. ProtParam analysis revealed that the sequence consists of 555 amino acids, with a molecular weight of 61056.28 Da and a molecular formula of C_2738_H_4347_N_781_O_741_S_31_. The theoretical isoelectric point is 10.89, with 13 negatively charged residues (Asp + Glu) and 64 positively charged residues (Arg + Lys). The aliphatic index of 78.34 indicates the protein’s thermostability, as a higher aliphatic index correlates with better thermal stability. After combining the 5′UTR and 3′UTR, the predicted translated protein of the mRNA vaccine had an average hydrophilicity index of −0.196, indicating it is a hydrophilic protein. The predicted secondary structure (Fig. 1A) and tertiary structure (Fig. 1B) include 4.32% β-turns, 18.92% α-helices, 54.23% random coils, and 22.52% extended strands. Using RNAfold software, we predicted the mRNA structure post-transcription. Figure 1C shows the optimal secondary structure with the minimum free energy (MFE) of −592.30 kcal/mol. Figure 1D presents another stable secondary structure with an MFE of −623.22 kcal/mol. The smaller the MFE, the more stable the structure, indicating that less energy is required to alter this structure. Immunogenicity is the ability to stimulate adaptive and cell-mediated immune responses, whereas antigenicity is the ability to be recognized by specific antigen molecules. Therefore, an appropriate vaccine candidate must exhibit both immunogenicity and antigenicity. Using the IDEB class I immunogenicity tool, we found the vaccine’s immunogenicity score to be 17.89073, indicating a strong capacity to stimulate immune responses. The developed multi-epitope vaccine also demonstrated antigenicity, with an antigenicity score of 0.904767 predicted by the ANTIGENpro server and 0.4229 estimated by the VaxiJen v2.0 server (scores above 0.4 are considered antigenic). Furthermore, the AllergenFP v.1.0 and AllerTOP v.2.0 servers confirmed that the candidate vaccine is non-allergenic to humans. Predicting the physicochemical properties of the vaccine is crucial for evaluating its efficacy and safety.

### Construction of pET-28a(+)/RP1C_1079–1518_ and prokaryotic expression, purification, and identification of recombinant protein RP1C_1079–1518_

**Fig 2:**
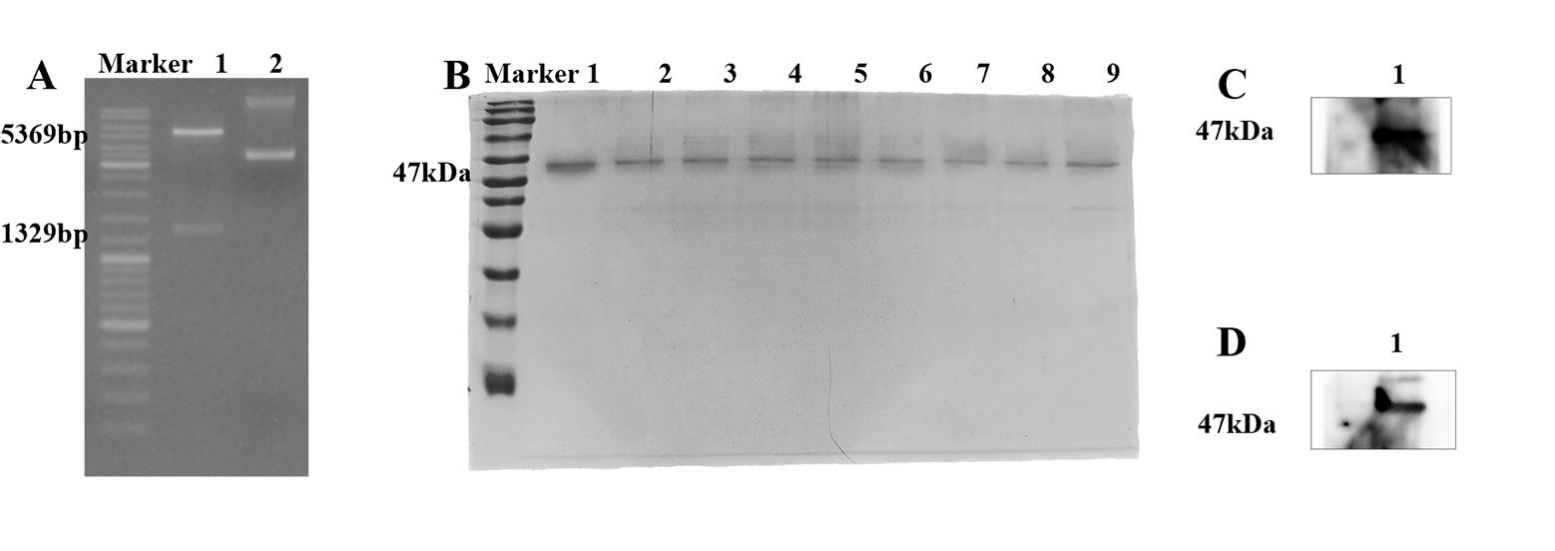
Construction, expression, purification, and identification of pET-28a(+)/RP1C1079–1518. (A) 0.8% agarose gel electrophoresis: Lane 1 shows the double digestion electrophoresis, and Lane 2 shows the original plasmid electrophoresis. (B) Coomassie brilliant blue analysis of RP1C1079–1518 separated by 12.5% SDS-PAGE: Lanes 1–9 represent RP1C_1079–1518_ eluted with 80 mM imidazole wash buffer, with an expected size of approximately 47 kDa. (C) Western blot analysis of *M. pneumoniae* RP1C_1079–1518_ using anti-*M*. *pneumoniae* antibody. (D). Western blot analysis of *M. pneumoniae* RP1C_1079–1518_ using anti-His antibody.

The expression vector pET-28a(+)/RP1C_1079–1518_ was constructed using the restriction enzymes BamHI and XhoI for double digestion. The successful construction of the vector was confirmed by DNA agarose gel electrophoresis (Fig. 2A), which showed a vector fragment of 5,369 bp and a target fragment of 1,329 bp. This indicated the successful construction of the prokaryotic expression vector. The recombinant protein RP1C_1079–1518_ was successfully expressed and purified in *Escherichia coli* BL21(DE3). The purity of the protein was assessed by SDS-PAGE analysis (Fig. 2B). To verify the expression of RP1C_1079–1518_, a Western blot experiment was performed. Initially, detection with anti-*M*. *pneumoniae* antibodies showed specific protein bands (Fig. 2C). Subsequently, detection with anti-His tag monoclonal antibodies (Fig. 2D) further confirmed the expression of RP1C_1079–1518_, as the recombinant protein contains His tags, allowing specific detection by anti-His antibodies. These results confirm the successful expression and purification of RP1C_1079–1518_ in a prokaryotic system, laying the groundwork for subsequent research.

### Construction and linearization of the cloning vector pGEM-3zf(+)/RP1C_1079–1518_

**Fig 3:**
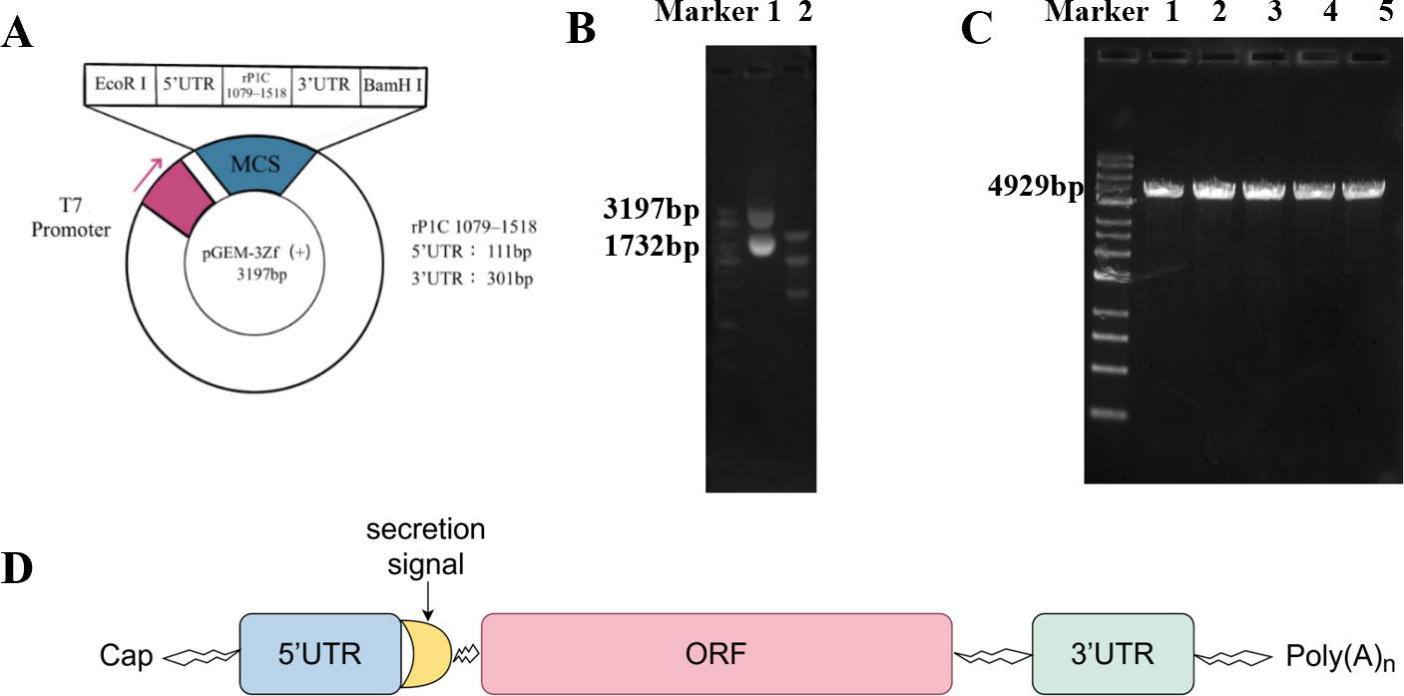
Construction and linearization of pGEM-3zf(+)/RP1C1079–1518. (A) Schematic representation of the pGEM-3zf(+)/RP1C_1079–1518_ cloning vector. (B) 0.8% agarose gel electrophoresis: Lane 1 shows the original plasmid electrophoresis, and Lane 2 shows the double digestion electrophoresis. (C) 0.8% agarose gel electrophoresis: Lanes 1–5 show the single digestion electrophoresis. (D) Sequence composition of mRNA vaccine.

The vector was digested using the restriction enzymes BamHI and EcoRI. The digestion products were analyzed by DNA agarose gel electrophoresis, revealing a vector fragment of 3,197 bp (Fig. 3A) and a target fragment of 1,732 bp (Fig. 3B). These results confirm the successful construction of the prokaryotic expression vector. The cloning vector was further digested using BamHI alone. Post-digestion analysis showed a plasmid DNA fragment of approximately 4,929 bp (Fig. 3C), indicating the successful linearization of the pGEM-3zf(+)/RP1C_1079–1518_ vector. This linearized DNA sequence was then transcribed *in vitro* and capped and tailed using the APE ×BIO kit (Fig. 3D).

### Preparation and characterization of LNP/mRNA

**Fig 4:**
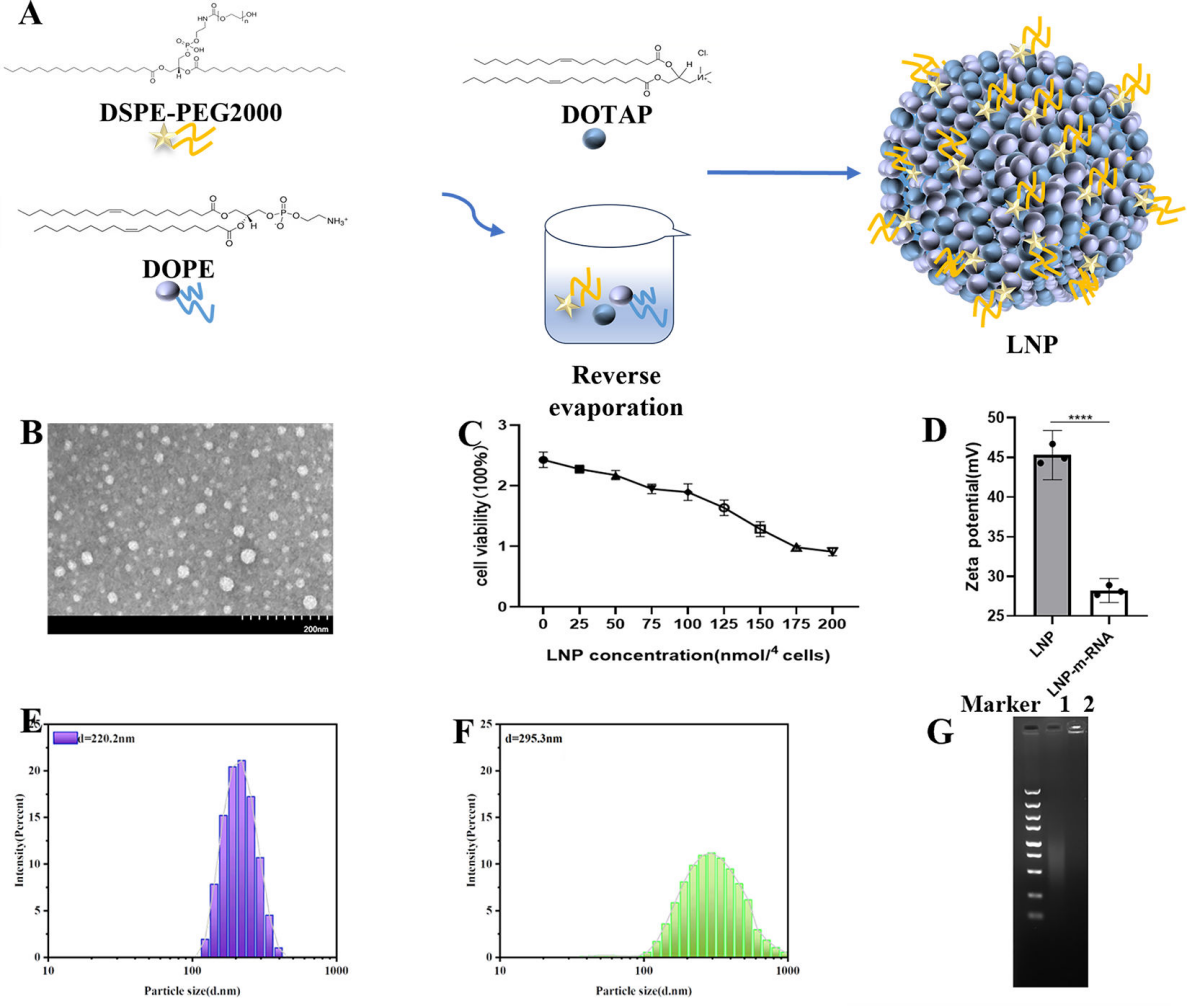
Preparation and characterization of LNP/mRNA. (A) Schematic diagram of LNP synthesis. (B) Transmission electron microscopy image showing the morphology of LNP particles. (C) Cytotoxicity assay of LNP. (D) Zeta potential measurement of LNP and LNP-mRNA. (E) Dispersion and particle size of LNP. (F) Dispersion and particle size of LNP-mRNA. (G) Gel retardation assay results: Lane 1 shows the mRNA group; Lane 2 shows the LNP-mRNA group.

The LNP was prepared with a molar ratio of DOTAP:DOPE:DSPE-PEG2000 (mol/mol) =50:50:1 (Fig. 4A). To understand the particle morphology of the LNP, transmission electron microscopy (TEM) was used for observation. As shown in Fig. 4B, the particles appeared approximately spherical. To determine a safe dosage for *in vitro* cell experiments and avoid cytotoxicity, a CCK-8 assay was performed (Fig. 4C). The results indicated that when the molar amount of nitrogen (N) in the material was ≤100 nmol per 10^4^ cells, the cell viability remained above 78%. However, at 150 nmol per 10^4^ cells, nearly 50% of the cells died, suggesting that this dosage could be problematic for cell experiments. Therefore, the dosage for subsequent *in vitro* cell experiments was controlled at around 100 nmol per 10^4^ cells. To assess changes in particle size and zeta potential before and after mixing the prepared LNP with mRNA, a Malvern Zetasizer Nano ZS90 was used. The average diameter of LNP was 220.2 d.nm (Fig. 4E) with a zeta potential of 45.3 mV (Fig. 4D). After combining with mRNA, the average diameter of LNP-mRNA increased to 295.3 d.nm (1.34 times larger) (Fig. 4F), and the zeta potential decreased to 28.2 mV (0.62 times lower) (Fig. 4D). The positive zeta potential of LNPs, primarily composed of cationic DOTAP, indicates a net positive charge on the material’s surface. Conversely, mRNA, which carries a negative charge, has a negative zeta potential. These results suggest that the combination of LNP with mRNA occurs through electrostatic interactions, leading to an increase in the particle diameter and a reduction in zeta potential. During transfection, LNP-mRNA can be electrostatically adsorbed onto the negatively charged cell membrane, facilitating mRNA entry into the cells. To evaluate the binding affinity between the lipid nanoparticle and mRNA, a gel retardation assay was performed. As shown in Fig. 4G, the mRNA not mixed with LNP migrated during agarose gel electrophoresis, displaying diffuse bands. By contrast, the LNP-mRNA complex remained entirely in the loading wells, indicating a strong binding affinity between LNP and mRNA, effectively hindering mRNA migration.

### *In vitro* transfection of mRNA and LNP-mRNA in eukaryotic cells

**Fig 5:**
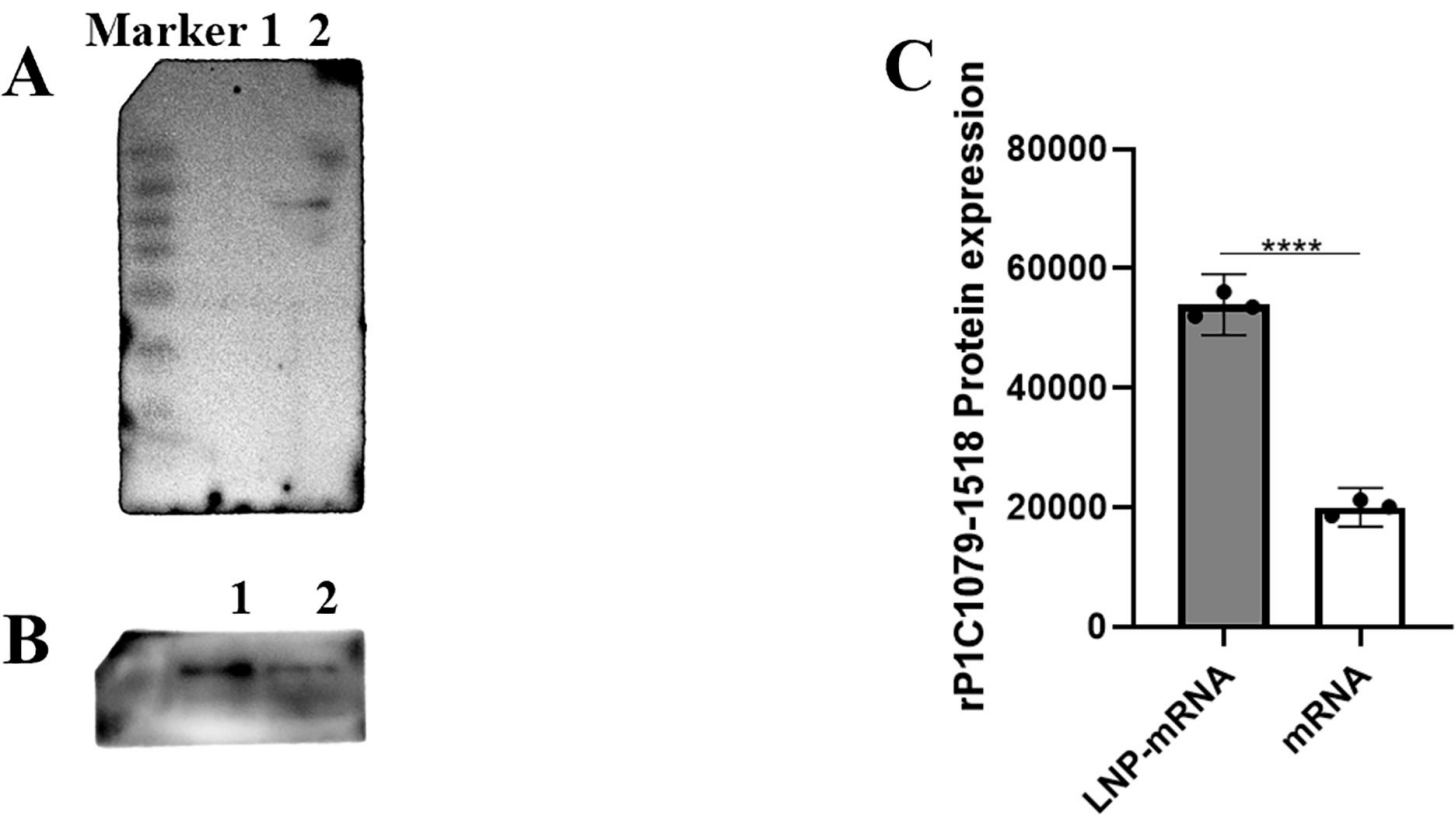
Western blot analysis of mRNA and LNP-mRNA expression in transfected A549 Cells. (A) Western blot analysis of mRNA transfection: Lane 1 shows the mRNA UTRs transfection group; Lane 2 shows the mRNA RP1C_1079–1518_ transfection group. (B) Western blot analysis of mRNA and LNP-mRNA transfection: Lane 1 shows the LNP-mRNA transfection group; Lane 2 shows the mRNA transfection group. (C) Densitometric analysis of Western blot results for mRNA and LNP-mRNA transfection. Data represent three independent experiments (*n* = 3). *P < 0.05, **P < 0.01, ***P < 0.001, ****P < 0.0001.

To determine whether the mRNA could be biologically functional and translate the RP1C_1079–1518_ protein in eukaryotic A549 cells after *in vitro* transfection, we conducted a Western blot analysis. Total protein from the transfected cells was analyzed using an anti-*M*. *pneumoniae* antibody as the primary antibody and HRP-conjugated goat anti-rabbit IgG as the secondary antibody. As shown in Figure 5A, a specific band corresponding to the RP1C_1079–1518_ protein (47 kDa) was observed in the experimental group. By contrast, no specific bands were detected in the negative control group (mRNA UTRs), indicating that the transfected mRNA could be translated into the endogenous antigen RP1C1079–1518 protein in eukaryotic A549 cells. To evaluate the transfection efficiency of LNP, A549 cells were transfected with LNP-mRNA, and non-encapsulated mRNA was used as a control. Total protein samples were extracted and analyzed by Western blot to determine whether the LNP-mRNA could translate the target antigen RP1C_1079–1518_ protein in cells. As shown in Figure 5B and C, the LNP-mRNA group exhibited a stronger ability to translate and express the RP1C_1079–1518_ protein compared to the non-encapsulated mRNA group, aligning with our expectations.

### Mice immunization and vaccine biosafety

**Fig 6:**
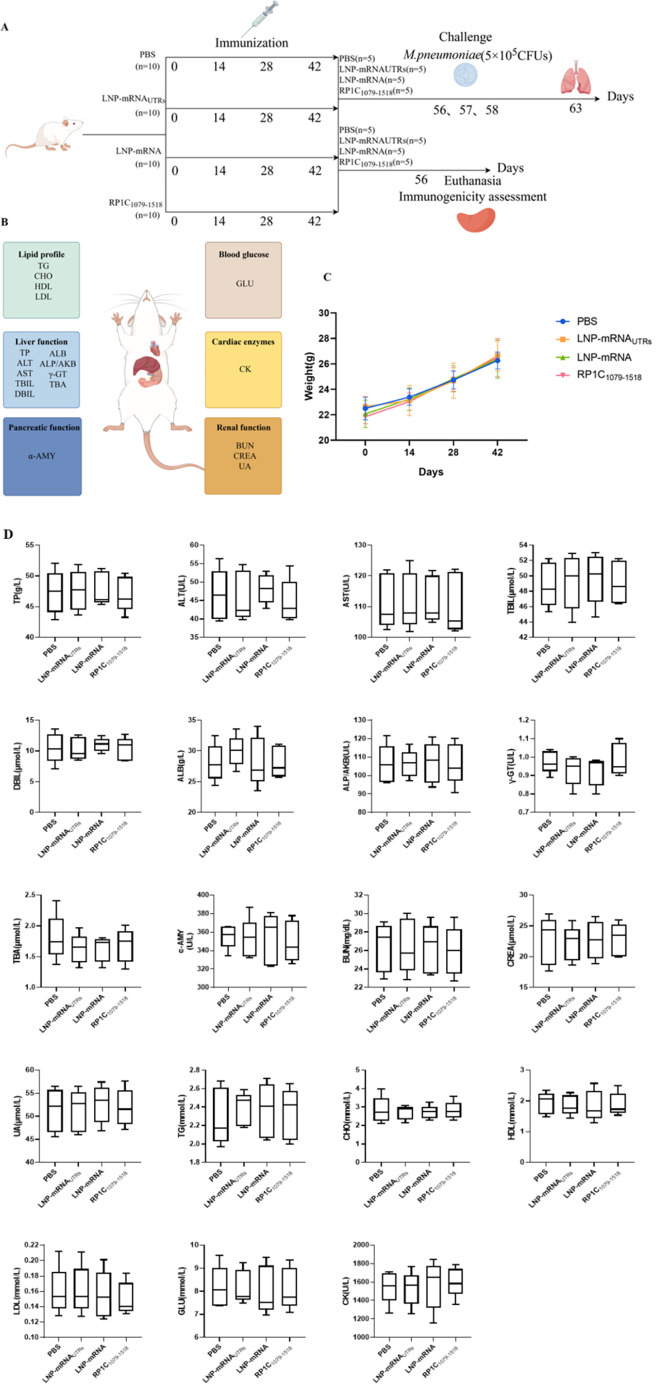
Mice immunization and vaccine biosafety. (A) Schematic diagram of the mice immunization schedule. (B) Schematic representation of blood biochemical test indicators in mice. (C) Body weight of mice before each immunization. (D) Blood biochemical test results of mice. In each case, the results are expressed as the average ±SD of the five replicate samples.*P < 0.05, **P < 0.01, ***P < 0.001, ****P < 0.0001.

To evaluate the biosafety of the designed vaccine (Fig. 6A), mice were immunized on days 0, 14, 28, and 42. Body weights were recorded before each immunization, and no significant differences were observed between the immunized group and the negative control group (Fig. 6C). Following standard clinical laboratory tests, the immunized group showed no significant differences compared to the negative control group in terms of the liver function, the kidney function, blood glucose, blood lipids, cardiac enzymes, and pancreatic function biomarkers. All measured values were within the normal range (Fig. 6B and D). The range of normal values for all biochemical tests is shown in Table 1.

**TABLE 1:** Normal value range for biochemical tests

Index		Unit	Range
Liver function	TP	g/L	38.02–75.06
	ALT	U/L	10.06–96.47
	AST	U/L	36.31–235.48
	T-BIL	μmol/L	6.09–53.06
	D-BIL	μmol/L	0.45–33.89
	ALB	g/L	21.22–39.15
	ALP	U/L	22.52–474.35
	γ-GT	U/L	0–7.78
	TBA	μmol/l	0–8.51
Pancreatic function	α-AMY	U/L	100–1000
Renal function	BUN	mg/dL	10.81–34.74
	CREA	μmol/L	10.91–85.09
	UA	μmol/L	44.42–224.77
Lipid profile	TG	mmol/L	0.84–2.72
	CHO	mmol/L	2.05–4.16
	HDL	mmol/L	1.28–2.65
	LDL	mmol/L	0.12–0.26
Blood glucose	GLU	mmol/L	4.66–13.42
Cardiac enzyme	CK	U/L	58.24–530.38

### Vaccine-induced specific antibody response and splenic lymphocyte proliferation in BALB/c mice

**Fig 7:**
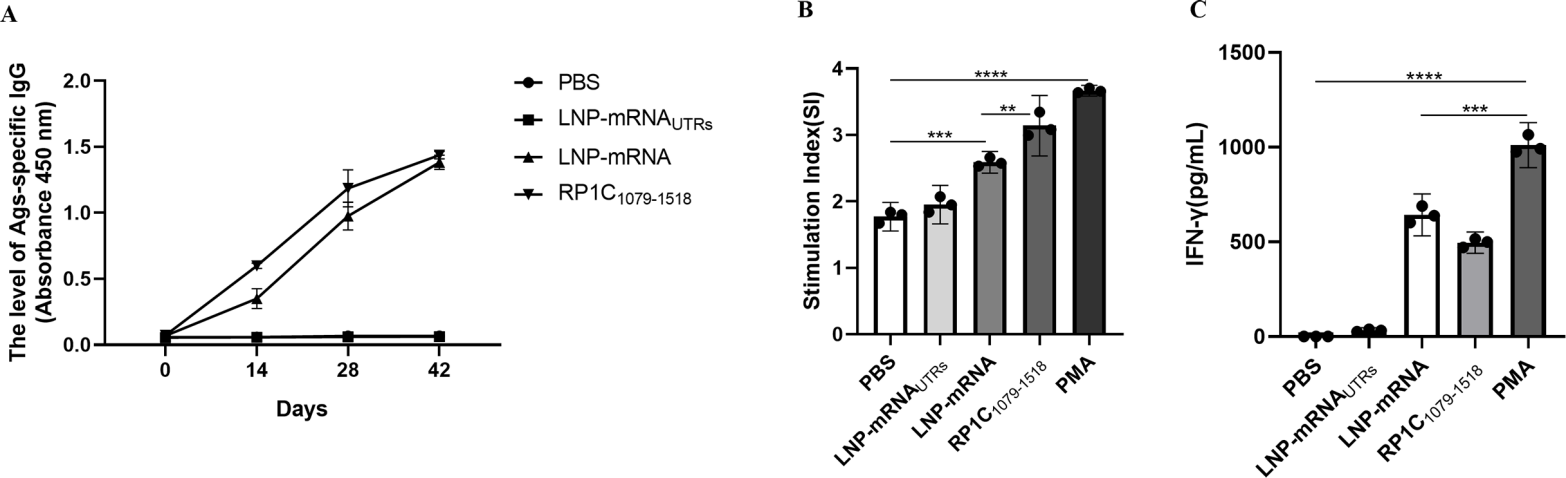
Vaccine-induced specific antibody response and splenic lymphocyte proliferation in BALB/c mice. (A) The level of Ags-specific IgG in mice serum from different groups post-immunization. (B) Stimulation index of splenic lymphocyte proliferation in mice. (C) Levels of IFN-γ in the mice spleen. Data are presented as mean ± 95% CI. Statistical significance tested by one-way ANOVA test (*P < 0.05; **P < 0.01; ***P < 0.001; ****P < 0.0001).

Using ELISA, we measured the titers of RP1C_1079–1518_ protein-specific IgG antibodies at different time points post-immunization with PBS, LNP-mRNA UTRs, LNP-mRNA, and RP1C_1079–1518_. As shown in Figure 7A, the LNP-mRNA and RP1C_1079–1518_ immunized groups exhibited a significant increase in specific IgG antibody levels by the second week, with titers peaking in the sixth week. To evaluate the specific cellular immune response, splenic T-lymphocyte proliferation was analyzed using the CCK-8 assay. The SI was compared among the groups. As depicted in Figure 7B, there was no significant difference between the PBS and LNP-mRNA UTRs groups. However, both the LNP-mRNA and RP1C_1079–1518_ groups showed significant differences, with the LNP-mRNA group displaying a slightly higher SI than the RP1C_1079–1518_ protein group. These results indicate that both the RP1C_1079–1518_ protein and LNP-mRNA groups possess strong T-lymphocyte proliferative capabilities. Figure 7C shows the IFN-γ levels in mice after different treatments. Compared with the PBS control group, LNP-mRNA was significantly increased, which was able to induce certain cellular immune responses.

### Detection of splenic T lymphocyte subsets by flow cytometry

**Fig 8:**
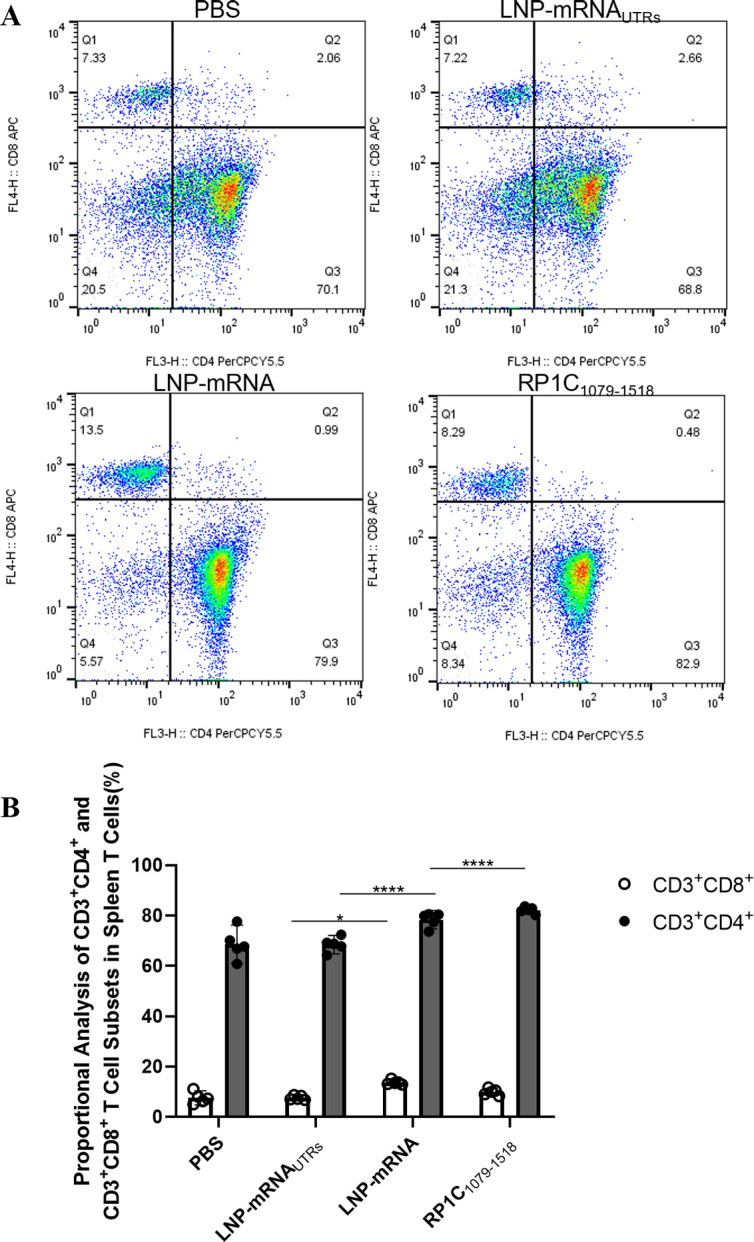
Flow cytometry analysis of T-lymphocyte subsets in spleen tissue. (A) Flow cytometry results of spleen tissue T-lymphocyte subsets. (B) Analysis of CD3^+^CD4^+^ and CD3^+^CD8^+^ T-lymphocyte levels. Data are presented as mean ± 95% CI. Statistical significance tested by one-way ANOVA test (*P < 0.05; **P < 0.01; ***P < 0.001; ****P < 0.0001).

On day 42, following the fourth immunization, half of the mice were euthanized. Spleen tissues were aseptically isolated and T-lymphocyte subsets were identified using flow cytometry. Data from each group were collected, organized, and statistically analyzed. The results, shown in Figure 8, indicate that the levels of CD3^+^CD4^+^ T lymphocyte subsets in the LNP-mRNA and RP1C_1079–1518_ groups were significantly different compared to the PBS and LNP-mRNA UTRs groups. Notably, there was a significant difference between the LNP-mRNA group and the PBS and LNP-mRNA_UTRs_ groups in the levels of CD3^+^CD8^+^T lymphocytes. However, no significant difference was observed between the LNP-mRNA group and the positive control group (RP1C_1079–1518_) in CD3^+^CD8^+^ levels. These results suggest that both the LNP-mRNA and RP1C_1079–1518_ groups can induce a strong CD4^+^ T-lymphocyte immune response. In addition, LNP-mRNA can induce a certain level of CD8^+^ T-lymphocyte immune response.

### Vaccine reduces pulmonary lesions and *M. pneumoniae* load and DNA copy numbers in BALB/c mice

**Fig 9:**
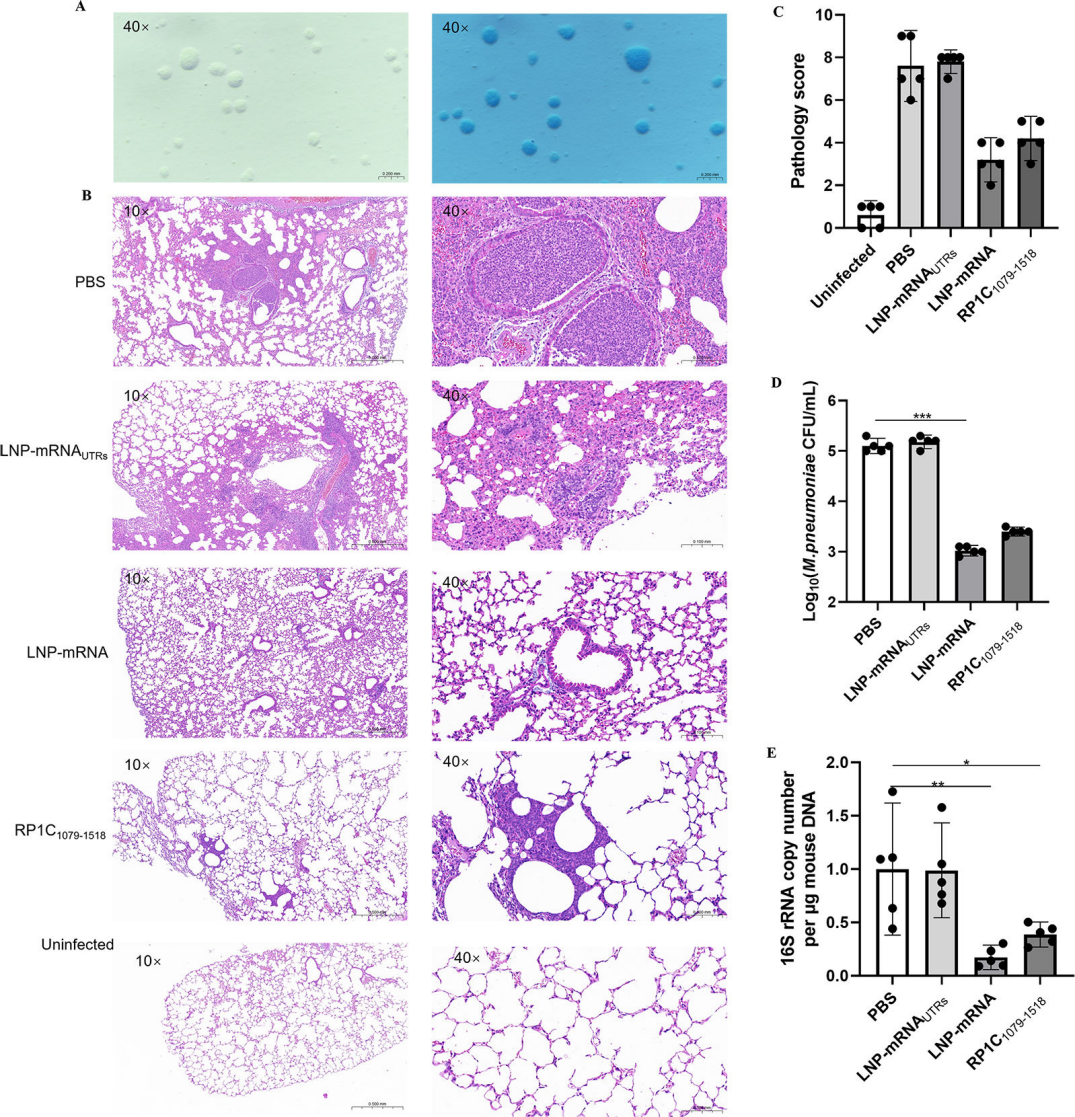
Detection of lung tissue changes in mice on the 7th day post *M. pneumoniae* challenge. (A) Unstained and Dienes-Stained *M. pneumoniae* on PPLO Agar. (B) H&E-stained lung tissue sections of mice from different groups post *M. pneumoniae* challenge. (C) Pathology score of lung tissue sections from mice. (D) *M. pneumoniae* DNA load in lung tissue homogenates of mice. (E) *M. pneumoniae* DNA copy numbers. Data are presented as mean ± 95% CI. Statistical significance tested by one-way ANOVA test (*P < 0.05; **P < 0.01; ***P < 0.001; ****P < 0.0001).

The standard strain of *M. pneumoniae*, M129 (ATCC 29342), was recovered from cryopreservation in a culture vial and subsequently inoculated onto a PPLO agar medium. After a 7-day incubation period, the colonies, exhibiting a characteristic fried-egg appearance, were observed under a microscope in both unstained and Dienes-stained preparations. The successful cultivation of *M. pneumoniae* was thereby confirmed (Fig. 9A). On the 7th day post-challenge, the lungs of mice were collected, paraffin-embedded, sectioned, and stained with HE. Pathological sections were randomly photographed at 10× and 40× magnification to compare lung pathology differences between the vaccine and control groups (Fig. 9B), and pathology scores were assessed (Fig. 9C). In the control groups (PBS and LNP), alveolar walls were thickened, alveolar spaces were reduced and unclear, and some alveoli were filled with red blood cells. Lesions, centered around the bronchioles, showed focal distribution and had merged into larger lesions in some areas. There was extensive inflammatory cell infiltration in the bronchial and alveolar spaces. In the vaccine group (LNP-mRNA), scattered lesions were also observed, with some bronchioles showing significant red blood cell presence. However, the number and area of lesions were significantly smaller than in the control group, and alveolar contours were clearer. On the 7th day post-challenge, mice were euthanized, and lung tissues were aseptically isolated. Lung tissue DNA was extracted and subjected to qPCR amplification for *M. pneumoniae* M129 16S rRNA and mouse β-actin genes to measure the *M. pneumoniae* DNA copy numbers in the lung tissue. Data from each group were collected and statistically analyzed. Figure 9D demonstrates the logarithmic values of colony forming units (CFU/mL) of *M. pneumoniae* in different groups of mice. The results showed that the number of *M. pneumoniae* was significantly reduced in the LNP-mRNA group and the RP1C1079–1518 group compared with the PBS group and the LNP-mRNA_UTRs_ group, especially in the LNP-mRNA group, and was significantly different from the PBS group. This suggests a potential protective effect of the LNP-mRNA vaccine in reducing *M. pneumoniae* lung infections. The results, as shown in Figure 9E, indicate that the *M. pneumoniae* DNA copy numbers in the lungs of the LNP-mRNA and RP1C_1079–1518_ groups were significantly lower than that in the PBS group. Notably, the *M. pneumoniae* DNA copy numbers in the LNP-mRNA and RP1C_1079–1518_ groups were significantly lower than in the LNP-mRNAUTRs group. These results suggest that the LNP-mRNA vaccine is effective in reducing pulmonary *M. pneumoniae* DNA copy numbers and alleviating lung lesions.

### Vaccine modulates IL-6, IL-4, IL-10, and IFN-γ production in BALB/c mice

**Fig 10:**
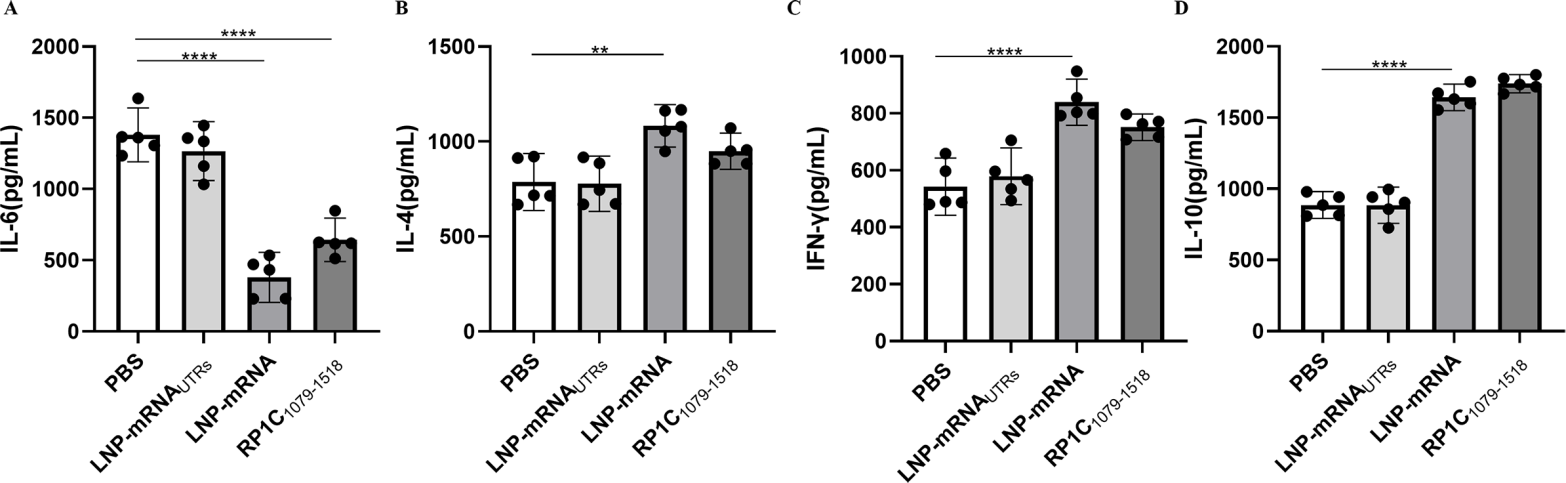
Cytokine detection in lung tissue homogenate supernatants post-*M. pneumoniae* challenge. (A) Detection of IL-6 in lung tissue homogenate supernatants. (B) Detection of IL-4 in lung tissue homogenate supernatants. (C) Detection of IFN-γ in lung tissue homogenate supernatants. (D) Detection of IL-10 in lung tissue homogenate supernatants. Data are presented as mean ± 95% CI. Statistical significance tested by one-way ANOVA test (*P < 0.05; **P < 0.01; ***P < 0.001; ****P < 0.0001).

On the 7th day post-challenge, mice from each group were euthanized, and lung tissues were aseptically isolated. The levels of cytokines IL-6, IL-4, IL-10, and IFN-γ in lung tissue homogenates were measured using a sandwich ELISA method. As shown in Figure 10B, C and D, the levels of IL-4, IL-10, and IFN-γ in the vaccine groups were significantly higher compared to the negative control group. IFN-γ, a Th1-associated cytokine, is involved in cellular immune responses (39). IL-4, a Th2-associated cytokine, stimulates B-cell proliferation and immunoglobulin production, thus participating in humoral immune responses. IL-10 exerts its anti-inflammatory properties by activating macrophages to inhibit the expression of inflammatory cytokines such as TNF-α, IL-6, and IL-1. In addition, IL-10 promotes B-cell proliferation, MHC class II antigen expression, and immunoglobulin secretion, and has a synergistic effect with IL-4 and IL-5 produced by Th2 cells (40–43). As depicted in Figure 10A, the IL-6 levels in the vaccine groups were significantly lower than in the negative control group. IL-6 can enhance the production of IL-17A by helper T cells, establishing a positive feedback loop for neutrophil recruitment and inflammation, thereby promoting inflammatory responses (23). These results indicate that the LNP-mRNA vaccine can induce the secretion of both Th1 and Th2 cytokines, leading to a balanced Th1-type cellular and Th2-type humoral immune response in immunized mice. In addition, the reduction in IL-6 levels suggests a decrease in inflammation, highlighting the vaccine’s ability to modulate immune responses effectively.

## DISCUSSION

The primary mechanisms by which *M. pneumoniae* causes disease in patients include adhesion to host cells, toxin release, and immune pathological reactions induced by *M. pneumoniae* (44, 45). Adhesion of *M. pneumoniae* to respiratory epithelial cells is the initial step in its pathogenesis, mediated by the adhesive organelles located at the tip of *M. pneumoniae*. Studies have shown that *M. pneumoniae* strains lacking adhesion capabilities also typically lack pathogenicity, indicating that adhesion is crucial for *M. pneumoniae* virulence (46). The adhesive organelles of *M. pneumoniae* are primarily composed of P1, P30, P40, P90, and high molecular weight proteins HMW1-3. Among these, P1 plays a major role as an adhesin. P1 is a multifunctional molecule that forms complexes with P30, P40, and P90, collectively localizing at the terminal organelle to perform various functions, such as receptor recognition and gliding motility. The P1 complex also includes P65, a truncated form of the P1C-terminus, and HMW proteins, which coordinate the adhesion of *M. pneumoniae* to host cells. Research indicates that P1 adhesin can directly bind to receptors on the surface of host cell membranes (47, 48). Its adhesive epitopes are located at the carboxyl terminus, exhibiting strong immunogenicity and directly inducing pulmonary inflammation (49, 50). Numerous studies have identified the C-terminus of *M. pneumoniae* P1 adhesin as a potential target for vaccine development. Adhesion inhibition experiments have demonstrated the presence of adhesion epitopes at the P1C terminus, confirming its adhesion function and suitability as a target peptide segment for vaccine development (51).

In recent years, bioinformatics have been extensively applied in biologic. Proteins are predicted using bioinformatics tools to lay the foundation for studying their functions. In this study, the C-terminal domain of P1 adhesin was analyzed for structure, antigenicity, and allergenicity using SMART, ANTIGENpro, VaxiJen v2.0, AllergenFP v1.0, and AllerTOP v2.0. Allergenic sequences were screened out, retaining extracellular sequences with high G + C content and important functional domains, such as those antagonizing ROS to reduce oxidative damage, participating in the regulation of gene expression transcription, and promoting the formation of native conformations during protein folding. mRNA vaccines are composed of a 5′Cap, 5′UTR, ORF, 3′UTR, and poly(A) tail. The selected advantageous antigenic peptide segment, identified through bioinformatics analysis, was used as the target sequence (ORF) and linked with UTRs. A set of UTRs that are stable and conducive to high expression was selected. In the design of an anti-mouse tumor vaccine in *Nature Medicine*, the firefly luciferase sequence was chosen as the UTR to effectively enhance the stability and translation efficiency of the mRNA vaccine (52). Our selection of UTRs with fewer target sites effectively avoids potential miRNA-induced translational inhibition. The target sequence was combined with UTRs, and an unstructured sequence segment called Kozak was inserted between the target sequence and the 5′UTR to significantly enhance translation efficiency, proportional to the inserted length. Bioinformatics prediction and analysis of the mRNA sequence were performed. SOPMA software was used to analyze the secondary structure of the target sequence, revealing that the secondary structure was primarily composed of α-helices and random coils, conducive to structural stability. RNAfold WebServer software was used to analyze the mRNA secondary structure, showing that the mRNA combined with the selected UTRs required the least free energy, indicating that the lower the required free energy, the more stable the structure and the higher the translation efficiency. Therefore, the 5′UTR selected human α-globin A1 (HBA1) and human hemoglobin A2 (HBA2) sequences, incorporating the Kozak consensus sequence for translation initiation in eukaryotic cells, followed by a secretion signal peptide. The P1 adhesin C-terminal adhesion epitope (amino acids 1,079–1,518) was selected as the mRNA coding region, with the stop codon TGA. The 3′UTR combined sequences from human AES/TLE5 and mitochondrial 12S RNA (mtRNR1). In addition, TGA at amino acids 1,098 and 1,159 of the P1 adhesin C-terminus was mutated to TGG, forming the final mRNA sequence.

mRNA molecules are inherently unstable and susceptible to degradation by nucleases. In addition, due to their charged nature, mRNA molecules have difficulty crossing the cell membrane to enter cells (53). LNPs are composed of various lipid components, including cationic or ionizable lipids, cholesterol, helper phospholipids, and polyethylene glycol (PEG)-lipids. The cationic or ionizable lipids interact electrostatically with the negatively charged mRNA molecules to form nanoparticles. As a delivery system, LNP effectively protects RNA molecules from degradation and promotes their entry into host cells (54, 55). Therefore, we chose to encapsulate mRNA in lipid nanoparticles to enhance the vaccine’s stability, delivery efficiency, and biocompatibility.

Studies have shown that children with MPP exhibit reduced CD4^+^ T lymphocytes, and adults infected with *M. pneumoniae* also show a decline in CD4^+^ T lymphocytes, possibly due to the involvement of these cells in the inflammatory response (56). This observation supports our experimental findings that the level of CD4^+^ T cells in the LNP-mRNA vaccine group was significantly higher than that in the PBS and LNP-mRNA_UTRs_ groups, which may be associated with the activation of Th1 and Th2 cells induced by the vaccine. The significant increase in CD8^+^ T cells indicates that the vaccine can activate cytotoxic T cells, which is crucial for clearing infected cells. These results are consistent with the expected effects of vaccine-induced immune responses, which provide protective immunity by activating various T-cell subsets.

In addition, the vaccine can regulate the production of cytokines such as IL-6, IL-4, IL-10, and IFN-γ, suggesting that it can induce a balanced Th1/Th2 immune response and may reduce inflammatory responses by lowering IL-6 levels. These findings are in line with current trends in vaccine research, which aim to optimize immune responses by modulating cytokine levels (57). The level of CD4^+^ T cells in the LNP-mRNA vaccine group was significantly higher than that in the PBS and LNP-mRNA_UTRs_ groups, which may be associated with the activation of Th1 and Th2 cells induced by the vaccine. The significant increase in CD8^+^ T cells indicates that the vaccine can activate cytotoxic T cells, which is crucial for clearing infected cells. These results are consistent with the expected effects of vaccine-induced immune responses, which provide protective immunity by activating various T-cell subsets. In addition, the vaccine can regulate the production of cytokines such as IL-6, IL-4, IL-10, and IFN-γ, suggesting that it can induce a balanced Th1/Th2 immune response and may reduce inflammatory responses by lowering IL-6 levels. These findings are in line with current trends in vaccine research, which aim to optimize immune responses by modulating cytokine levels.

Differences in antigen recognition pathways can lead to the differentiation of CD4^+^ T cells into various T-cell subtypes, each producing distinct cytokines that play different roles in the anti-infection process. Research has shown that Th1-type cells produce significant amounts of pro-inflammatory cytokines, particularly IFN-γ, which is beneficial for clearing infections related to viruses and mycoplasmas. In addition, IFN-γ can suppress inflammation at the infection site by downregulating the Th2 immune response. If the Th2 response is not adequately downregulated, it may lead to a negative feedback loop, exacerbating the consumption of IFN-γ (58). In this study, we observed that on the 10th day post-infection, the IL-6 levels in the lung homogenates of the LNP-mRNA group were significantly lower than those in the negative control group. Notably, the *M. pneumoniae* DNA copy numbers in the lungs of the LNP-mRNA group were also significantly lower than that of the negative control group. These results suggest that the LNP-mRNA vaccine may facilitate the clearance of *M. pneumoniae* infection by jointly inducing Th1 and Th2 immune responses.

In vaccine-induced immune responses, the increase in IL-4 may indicate the activation of a Th2 immune response, which aids in the activation of B cells and the production of antibodies, thereby participating in the humoral immune response. However, an excessive increase in IL-4 may also be associated with the exacerbation of inflammatory responses (59). For instance, during acute asthma attacks, IL-4 can significantly increase the aggregation and activation of inflammatory cells in the airways, thereby inducing and aggravating acute asthma episodes (60). Furthermore, monocytes and B cells are the primary sources of IL-10 in humans (61). IL-10 exerts its anti-inflammatory properties by activating macrophages to suppress the expression of inflammatory cytokines such as TNF-α, IL-6, and IL-1 (62). In addition, IL-10 promotes the proliferation of B cells, the expression of MHC class II antigens, and the secretion of immunoglobulins, and it has a synergistic effect with IL-4 and IL-5 produced by Th2 cells (63).

However, the specific mechanisms remain unclear and warrant further investigation. The interaction between mycoplasma and the host immune system is evidently complex. Therefore, it is essential to monitor all aspects of the immune response during each vaccine experiment, particularly at the cellular and molecular levels. By analyzing, summarizing, and inferring from existing data, we can determine the precise mechanisms by which the vaccine induces immune protection. This will help in further refining and optimizing vaccine design to develop more specific, safe, and effective vaccines against *M. pneumoniae* infection. To better understand and evaluate the unique immunomodulatory effects of LNP, further research using T-cell analysis and transcriptomic studies is necessary.

In our research, we employed splenocytes to evaluate T-cell responses, a method that is routinely and widely recognized in immunology. Nevertheless, we recognize the pivotal role that mediastinal lymph nodes play in the immune response to respiratory infections, especially when assessing T-cell responses induced by vaccines targeting lung infections (64). Due to their proximity to the lungs, mediastinal lymph nodes can rapidly capture and present antigens to T cells, thereby triggering effective local immune responses, which may provide more accurate and specific information for vaccine-induced immune responses. Further studies on the role of mediastinal lymph nodes in vaccine-induced immune responses could offer deeper insights, which are crucial for optimizing vaccine designs to effectively target respiratory infections. We believe that despite this limitation, our study still provides valuable insights and data. We are committed to overcoming these constraints in future research and to exploring more comprehensive methods for assessing immune responses, including the analysis of mediastinal lymph nodes.

We employed qPCR to assess *M. pneumoniae* DNA in lung homogenates, recognizing that this method cannot differentiate between viable bacteria and bacterial remnants. Despite this limitation, qPCR’s high sensitivity made it suitable for our comparative analysis, as it detects low bacterial levels that culture methods might miss. While bronchoalveolar lavage fluid (BALF) could provide a direct measure of viable bacteria, our use of lung homogenates captures a more comprehensive infection profile, including intracellular bacteria. This method was chosen for its ability to reveal the overall bacterial burden within the lung, which is essential for understanding the pathogen’s systemic impact. Future studies could benefit from incorporating BALF analysis to directly assess viable bacterial loads, complementing our current findings.

In addition to the design of the target mRNA sequence and the choice of the LNP delivery system, the route of immunization also significantly impacts the immunoprotective efficacy of mRNA vaccines. Literature indicates that the delivery methods for vaccines can be categorized into three major types: systemic delivery, mucosal delivery, and combined systemic and mucosal delivery (65). In this study, due to the advantages of reduced local side effects, we utilized only intramuscular injection for immunizing mice. This route provided partial immune protection against *M. pneumoniae* pulmonary infection. Unfortunately, this study did not explore whether this route could elicit a mucosal immune response. Future studies should consider employing various immunization routes to administer the mycoplasma mRNA vaccine. This would help evaluate the effects of different immunization methods on mucosal immune responses and overall vaccine efficacy. Exploring these aspects could lead to optimized immunization strategies that enhance both systemic and mucosal immunity, ultimately improving the protective effectiveness of mycoplasma vaccines.

Due to the similarities in the development and progression of certain diseases between mice and humans, as well as the high biological and genetic similarity, mice are often chosen as surrogate models for research in disease prevention, drug, and vaccine development (66). A review of the literature reveals that most mycoplasma vaccine experiments are conducted using surrogate hosts. Although many mycoplasma vaccines have achieved success in surrogate animal models such as mice and guinea pigs, some vaccines have failed to replicate these results in non-human primate models (44). This discrepancy is likely due to inherent differences in interactions between mycoplasmas and different hosts. Given the differences in mycoplasma strains and host infections, some vaccine experiments lack comprehensiveness in the choice of animal models, limiting their applicability. The selection of appropriate animal models remains a significant challenge in mycoplasma vaccine research. To further evaluate the immunoprotective efficacy of *M. pneumoniae* mRNA vaccines, it is essential to consider more comprehensive animal models or to select the anticipated host of *M. pneumoniae* as the animal model. This approach would help assess the vaccine’s ability to induce cellular and humoral immune responses and the duration of immune function maintenance. In addition, differences in the genetic background of major histocompatibility complexes (MHC) (mouse, H-2; human, HLA) lead to variations in immune responses to antigens between mice and humans (67). Few studies have formally evaluated the differences in antibody evaluation indices and cytokine expression levels between mice and humans post-infection. Therefore, the effectiveness of vaccines should be objectively verified through clinical efficacy monitoring. In conclusion, while mouse models provide valuable insights, expanding the range of animal models to include more relevant hosts will be crucial for advancing mycoplasma vaccine research. Clinical trials will ultimately be necessary to validate the efficacy of mRNA vaccines for *M. pneumoniae* in humans, ensuring that these vaccines are both effective and safe for human use.

Long-term safety considerations are also critical, as potential adverse effects may only emerge over time. Comprehensive monitoring in both animal models and human trials is essential for ensuring the long-term safety and well-being of vaccinated individuals. The safety monitoring of the bivalent COVID-19 mRNA vaccine, for instance, has shown that most reports were nonserious and usually similar to those after first and second booster vaccinations. However, myocarditis and pericarditis, though rare, have been associated with mRNA vaccines (68). In conclusion, while the initial results from animal models are promising, the long-term safety of mRNA vaccines must be rigorously evaluated through extended follow-up studies and surveillance. This approach will provide the necessary data to ensure the safety and efficacy of these vaccines in the long term.

In this study, our research group explored the concept of an anti-*M*. *pneumoniae* vaccine based on adhesion epitopes rather than using the entire pathogen. In some cases, a large number of unnecessary antigens can trigger allergic reactions. Therefore, epitope-based vaccines may be a favorable approach to reducing infection recurrence and eliciting a robust immune response. Epitopes are immune stimulants presented by MHC molecules on cells and are subsequently recognized by T or B cells. Both T and B cells cooperate with various immune cells that secrete cytokines, providing adaptive immunity to host cells (69). We employed several bioinformatics methods to lay the groundwork and provide theoretical support for developing a candidate vaccine capable of eliciting an effective immune response against *M. pneumoniae* infection. This vaccine is based on the adhesion epitopes of the P1 protein. The vaccine was found to possess both antigenic and immunogenic characteristics. In addition, the recombinant protein RP1C_1079–1518_ was used as a positive control group to comprehensively evaluate the endogenous antigen protein expression and exogenous antigen protein as vaccines in inducing protective immune responses in BALB/c mice. Our approach focused on utilizing specific adhesion epitopes of the P1 protein to minimize potential allergic reactions while maximizing the immunogenic response. The comprehensive evaluation of both endogenous and exogenous antigen proteins underscores the vaccine’s potential to induce protective immunity.

### Conclusion

The results of this study indicate that the *M. pneumoniae* mRNA vaccine provides a certain level of protection against challenge infection. Although the vaccine did not achieve 100% protection in mice, this may be related to the immunization dosage, schedule, and immune mode. Therefore, future studies should focus on optimizing the immunization dosage and schedule to enhance the protective efficacy of the vaccine. Overall, the designed mRNA vaccine was capable of inducing both humoral and cellular immune responses against *M. pneumoniae*, reducing pulmonary mycoplasma load and inflammatory responses. This study provides a potential approach for developing an effective *M. pneumoniae* vaccine. Our findings demonstrate that the *M. pneumoniae* mRNA vaccine has good biosafety, which is considered a fundamental quality for vaccines. However, this study has some limitations that need to be addressed in future research. For instance, adjusting the immunization dosage and schedule, exploring different routes of administration, and evaluating the long-term immunity and protection in more diverse and relevant animal models are crucial steps toward improving and validating the vaccine’s efficacy and safety. These future studies will be essential for advancing this vaccine candidate toward clinical application.
